# A Longitudinal Approach to Assessing Urban and Suburban Children’s Exposure to Pyrethroid Pesticides

**DOI:** 10.1289/ehp.9043

**Published:** 2006-04-26

**Authors:** Chensheng Lu, Dana B. Barr, Melanie Pearson, Scott Bartell, Roberto Bravo

**Affiliations:** 1 Department of Environmental and Occupational Health, Rollins School of Public Health, Emory University, Atlanta, Georgia, USA; 2 National Center for Environmental Health, Centers for Disease Control and Prevention, Atlanta, Georgia, USA

**Keywords:** children’s pesticide exposure, dietary exposure, PBA, permethrin, pyrethroids, residential exposure, *trans*-DCCA, urinary biomarker

## Abstract

We conducted a longitudinal study to assess the exposure of 23 elementary school–age children to pyrethroid pesticides, using urinary pyrethroid metabolites as exposure biomarkers. We substituted most of the children’s conventional diets with organic food items for 5 consecutive days and collected two daily spot urine samples, first morning and before bedtime voids, throughout the 15-day study period. We analyzed urine samples for five common pyrethroid metabolites. We found an association between the parents’ self-reported pyrethroid use in the residential environment and elevated pyrethroid metabolite levels found in their children’s urine. Children were also exposed to pyrethroids through their conventional diets, although the magnitude was smaller than for the residential exposure. Children’s ages appear to be significantly associated with pyrethroids exposure, which is likely attributed to the use of pyrethroids around the premises or in the facilities where older children engaged in the outdoor activities. We conclude that residential pesticide use represents the most important risk factor for children’s exposure to pyrethroid insecticides. Because of the wide use of pyrethroids in the United States, the findings of this study are important for both children’s pesticide exposure assessment and environmental public health.

Pyrethroids, a group of synthetic insecticides, were manufactured in the 1970s after the removal of organochlorine insecticides, such as DDT, from the consumer market. The synthetic pyrethroids not only inherit the biologic activity (ability to kill insects) from their natural counterpart, pyrethrin, which is found in chrysanthemums, but are also improved in their environmental stability. Pyrethroids are widely used in agriculture, forest, textile industry, and public health programs worldwide (Heudorf and Angerer 2001). With the phaseout of organo-phosphorus (OP) pesticide use in residential environments in the United States [U.S. Environmental Protection Agency (EPA) 1998], the availability of pyrethroids for consumer uses has increased since the late 1990s (U.S. EPA 2005).

Although individual pyrethroid insecticides share some common physical and chemical properties as a group, their toxicologic mechanisms, unlike those of OP pesticides, vary in mammals. Pyrethroid insecticides are subject for review as potential developmental neurotoxicants because of their mode of action on voltage-sensitive sodium channels (Kolaczinski and Curtis 2004; Shafer et al. 2005). In addition, permethrin, the most widely used pyrethroid insecticide, is suspected to be an endocrine-disrupting chemical (Chen et al. 2002; Kakko et al. 2004; Kim et al. 2004) and, along with fenvalerate, has been classified as a potential carcinogen at high exposure levels (U.S. EPA 2006). Toxicologic studies have also suggested that pyrethroids have a suppressive effect on the immune system and may cause lymph node and spleen damage (Repetto and Baliga 1997).

Although pyrethroids have been sold in the U.S. consumer market for > 30 years, with the estimated annual use ranging from several thousand to a million pounds (Agency for Toxic Substances and Disease Registry 2003), very few studies have been conducted to quantitatively assess human exposures to pyrethroids. Most of the relevant data were obtained from studies conducted in Germany or in occupational settings (Berger-Preiss et al. 2002; Hardt and Angerer 2003; Leng et al. 2003). Recently, the Centers for Disease Control and Prevention (CDC) reported urinary pyrethroid metabolite levels for the U.S. population 6–59 years of age in the Third National Report on Human Exposure to Environmental Chemicals, which is part of the National Health and Nutrition Examination Survey (NHANES) conducted in 2001–2002 (CDC 2005). All of these studies were conducted cross-sectionally, so the results represent exposures only over relatively short time periods.

The primary objective of this study was to establish a temporal profile of pyrethroid exposure in a cohort of elementary school–age children living in an urban/suburban community, using urinary pyrethroid metabolites as exposure biomarkers. We also examined the relationship between pyrethroid exposure and children’s diets, self-reported residential pyrethroid use, and age.

## Materials and Methods

This study is part of the Children’s Pesticide Exposure Study (CPES), which took place in the Seattle, Washington, area from July 2003 to May 2004. Details of the study design and methods have been published previously (Lu et al. 2006). In brief, 23 children 3–11 years of age were recruited from local public elementary and Montessori schools for a 15-consecutive-day sample period in summer 2003, with repeated samplings in fall 2003 and in winter and spring 2004. This report discusses results of urinary pyrethroid metabolites only for the summer 2003 sampling period. Results from other sampling periods will be reported when they become available. Subject eligibility for enrollment included children exclusively consuming conventional diets and spending most of their time in one residence. Household pesticide use information was obtained via an in-person interview during an in-home appointment before the field sampling. Written consent was obtained from parents and older children who can read the consent form, whereas oral assent was obtained from younger children. The University of Washington Human Subject Committee approved the use of human subjects in this study.

### Sampling period

As previously reported (Lu et al. 2006), the 15-consecutive-day sampling period was divided into three phases. During phase 1 (days 1–3) and phase 3 (days 9–15), children consumed their normal conventional diets. During phase 2 (days 4–8), organic food items, including fresh fruits and vegetables, juices, processed fruit or vegetables (e.g., salsa), and wheat- or corn-based items (e.g., pasta, cereal, popcorn, or chips), were substituted for the children’s conventional diet. These food items are routinely reported to contain pesticide residues [U.S. Department of Agriculture (USDA) 2003]. Meats and dairy products were not substituted.

### Urine sample collection and analysis

For 15 consecutive days, the first morning void and last void of the day were collected from each child. These urine samples were refrigerated or maintained on ice before processing in the lab and then stored at –20°C until pesticide metabolite analysis was performed in the National Center for Environmental Health at the CDC in Atlanta, Georgia, using a liquid chromatography/tandem mass spectrometry method (Olsson et al. 2004). The targeted metabolites for pyrethroids included 3-penoxy-benzoic acid (PBA), 4-fluoro-PBA (FPBA), *cis*-2,2-(dichloro)-2-dimethylvinylcyclopropane carboxylic acid (*cis*-DCCA), *trans*-DCCA, and *cis*-2,2-(dibromo)-2-dimethylvinylcyclopropane carboxylic acid (DBCA). Kuhn et al. (1999) demonstrated the relationship of chemical structures between common pyrethroids and their urinary metabolites. The limits of detection (LOD) for the metabolites of pyrethroids insecticides are listed in Table 1.

### Data analysis

To analyze the data, we used the reported concentrations for samples with detectable (> LOD) or detectable but not quantifiable (< LOD) levels, whereas “0” was assigned for samples where nothing was detected. We calculated the daily volume-weighted average (DVWA) of pyrethroids metabolites by averaging the metabolite concentration in the morning sample with the previous day’s bedtime sample normalized by the total volume of these two urine samples (Lu et al. 2006). In cases where only one of the two spot urine samples was collected, the metabolite concentration of the collected sample was used as the DVWA concentration. Urinary concentrations of pyrethroid metabolites were not adjusted by creatinine or specific gravity.

Because the pyrethroid urinary metabolite concentrations were skewed and subject to LODs, one-half of the LOD for each of the pyrethroid metabolites was added to all the respective measured urinary metabolite concentrations before log-transformation. Parametric tests were therefore used for statistical analyses using SPSS (version 11; SPSS Inc., Chicago, IL). We used a linear mixed-effects model to test the associations with diet and self-reported residential use of pyrethroid pesticides, which represents the primary risk factors for children’s exposure to pyrethroid pesticides. In addition, age was included in the expanded model to determine whether children’s age also influenced pyrethroid exposure.

## Results

The frequency of detection for the different urinary pyrethroid metabolites varied (Table 1). The most frequently detected pyrethroid metabolite was PBA, a nonspecific urinary metabolite for many pyrethroid insecticides (Heudorf and Angerer 2001) such as permethrin, cypermethrin, and deltamethrin, followed by *trans*- and *cis*-DCCA, two urinary metabolites for permethrin, cypermethrin, and cyfluthrin. Very few urine samples had detectable levels of FPBA and DBCA, specific metabolites for cyfluthrin and deltamethrin, respectively.

Table 2 shows the descriptive statistics of the DVWA concentrations for the five urinary pyrethroid metabolites. In parallel to the frequency of detection, PBA has the highest median DVWA level, followed by *trans*-DCCA. Because most of the urine samples had nondetectable levels for *cis*-DCCA, FPBA, and DBCA, their data were not included in statistical analyses. The concentration ratios of *cis*-and *trans*-DCCA, which range from 0.3 to 0.6, correspond to the isomer content of pyrethroid pesticides in most consumer products. For example, the content of permethrin in most commercial products contains either 1:3 or 2:3 of the *cis*- and *trans*-isomers.

**Figure 1:**
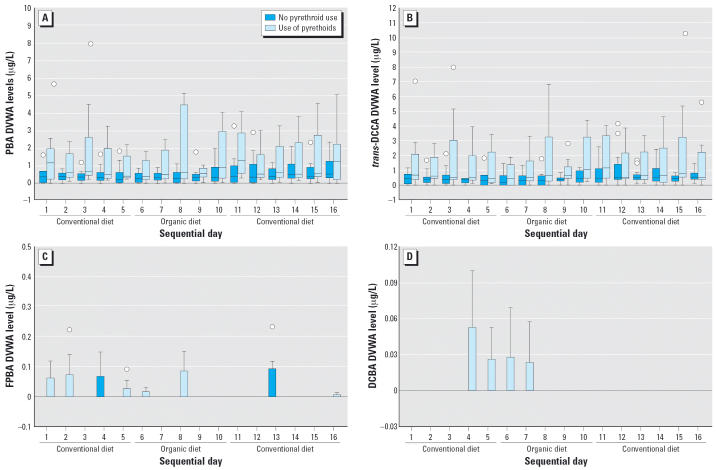
Box plots, separated by self-reported residential use of pyrethroid pesticides, of DVWA of pyrethroid concentrations in 23 children 3–11 years of age for 15 consecutive days in which conventional and organic diets were consumed: (*A*) PBA, (*B*) *trans*-DCCA, (*C*) FPBA, and (*D*) DBCA. In the box plots, the horizontal lines in each plot represent 10th, 25th, 50th, 75th, and 90th percentiles, bottom to top. Circles represent outlier values. Extreme values are not included in the plots.

We could identify no apparent trend due to the dietary intervention, as was seen for OP pesticides (Lu et al. 2006), on visual inspection of the box plots of the DVWA pyrethroid metabolites in the 23 children during the 15-day study period. Many urine samples collected during the organic diet period continued to have detectable levels of all five pyrethroid metabolites. However, when we grouped the children by whether or not pyrethroids were used in their household, we found that children of families who used pyrethroids had higher and more variable DVWA PBA and *trans*-DCCA levels than did those not using pyrethroids at home (Figure 1A,B). Although detection of FPBA and DBCA in this cohort was rare and the levels were generally low, it is evident that most of these exposures were associated with residential use of pyrethroids (Figure 1C,D).

Seven of the 23 families reported and were confirmed to use pyrethroid pesticides in their households, both inside and outside the homes (Table 3). Four of the seven families had an older child (≥8 years of age) who participated in this study. Urine samples collected from these seven children routinely contained detectable pyrethroid metabolite levels, and among those urine samples, several had the highest concentrations of pyrethroid metabo-lites. Most detectable FPBA and all the detectable DBCA levels in urine samples were collected from these seven children.

**Figure 2:**
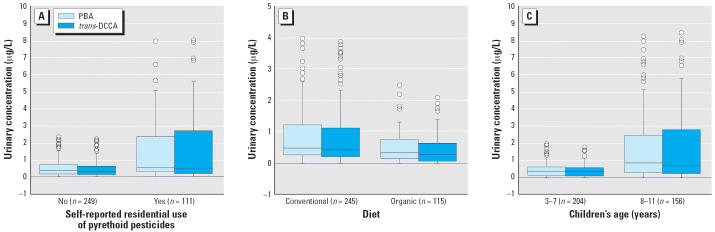
DVWA of PBA and *trans*-DCCA in 23 children 3–11 years of age for 15 consecutive days by self-reported residential use of (*A*) pyrethroid pesticides, (*B*) diet, and (*C*) age. In the box plots, the horizontal lines in each plot represent 10th, 25th, 50th, 75th, and 90th percentiles, bottom to top. Circles represent outlier values. Extreme values are not included in the plots.

Results from the linear mixed-effects model (Table 4) demonstrated that both residential pyrethroid use and diet are significant contributors to both PBA and *trans*-DCCA levels in the urine. It is evident from this model that the effect of self-reported residential pyrethroid use is more important than that of diet. Two separate one-way analyses of variance (ANOVAs) analyzing PBA and *trans*-DCCA data individually for diet or residential use of pyrethroids (Table 5) indicated that the median DVWA PBA and *trans*-DCCA levels are significantly higher in children whose parents reported use of pyrethroids at home than in those who did not (Figure 2A), and significantly higher during the conventional diet days than during the organic diet days (Figure 2B). The results from the expanded linear mixed-effects model suggested that, in addition to diet and residential pyrethroid use, children’s age is also a significant predictor of PBA and *trans*-DCCA levels in the urine (Table 6). The median DVWA PBA and *trans*-DCCA levels are significantly higher in older children (8–11 years of age) than in younger children (3–7 years of age) (Figure 2C).

## Discussion

Despite the wide use of pyrethroid pesticides, the assessment of pyrethroid pesticide exposure in the U.S. population, particularly for children, is limited to a handful of cross-sectional studies. This report consists of data collected as part of the ongoing CPES with a focus on children’s exposure to pyrethroid pesticides. This longitudinal data set provides a more complete documentation of children’s exposure to pyrethroids and examines potential risk factors for higher exposure levels.

The most significant finding of this study—the association between self-reported pyrethroids use in the residential environment by the parents and the elevated pyrethroid metabolite levels found in their children’s urine—is important for both children’s pesticide exposure assessment and environmental public health. Interventions could focus on minimizing the use of pyrethroids in the residential environment or eliminating the possible contact with treated areas or objects by children. This finding is consistent with those of a previous report suggesting that children whose parents reported residential OP pesticide use had higher OP urinary metabolite concentrations (Lu et al. 2001). Although changing children’s diets from conventional to organic food also lowers their exposures to pyrethroid pesticides, the effect of such dietary intervention is not as dramatic for pyrethroid pesticides as it is for OP pesticides (Lu et al. 2006). This pattern is in agreement with the general theory that children are exposed continuously to low levels of pesticides through their diets and that this chronic exposure is modified, usually increased, by episodes of relatively high exposures from other pathways, such as residential use (Shurdut et al. 1998).

We conclude that residential pesticide use represents a very important risk factor for children’s exposure to pyrethroid insecticides. This conclusion is supported by the fact that only seven of the 23 families reported residential pyrethroid use, yet such use accounted for more of the variability in the urinary pyrethroid metabolites than did the dietary intervention. The results from the mixed-effects model indicated that residential use of pyrethroids remains a more significant predictor of both urinary PBA and *trans*-DCCA when different diets were taken into account. The data presented in Table 3 clearly demonstrate that children who lived in households where pyrethroids have been used for pest control purposes have experienced much higher pyrethroid exposures than those whose parents reported no pyrethroid use in their homes. Four of the five highest pyrethroid metabolite levels were found in these seven children. An extreme case was that of a 4-year-old child whose parents used permethrin on the furniture, including beds. Several urine samples collected from this participant have the highest DVWA PBA and *cis*- and *trans*-DCCA levels. Other children who lived in homes where pyrethroids were used were continuously exposed to this group of insecticides throughout the 15-day study period. Notably, our pesticide use survey asked whether pesticides are used in and around the home and, if so, when the last application occurred. Thus the continuous exposure to these pesticides throughout the 15-day study period likely reflects residual sources.

Children were also exposed to pyrethroids through diets, although the magnitude was smaller than the residential exposure. Results from studies conducted in Germany have suggested that exposure to pyrethroids in the general population is caused by uptake with the diet (Heudorf and Angerer 2001; Schettgen et al. 2002); however, no direct evidence was provided to support this conclusion. In our study, all five of the measured pyrethroid metabolites were found during the organic diet period, including the two least frequently detected metabolites, FPBA and DBCA, specific metabolites for cyfluthrin and deltamethrin, respectively. Accordingly, most of the children’s exposures to pyrethroid metabolites are likely to have come from the environment.

In this study, age is a significant predictor for pyrethroid exposure. We found that older children, 8–11 years of age, experienced higher pyrethroid exposures than did children 3–7 years of age. This finding is not consistent with results from other studies, which suggest that younger children tend to have higher pesticide exposure than older children. Younger children are more vulnerable to adverse health risks resulting from pesticide exposures because of the difference in their physiologic functions relative to older children and adults. However, it is difficult to draw an absolute conclusion that younger children have higher pesticide exposures, particularly exposures from residential environment, than older children. Although common characteristics of young children, such as hand-to-mouth behaviors and close proximity to the floor, put them at higher risk of pesticide exposure, it is the pesticide residues found in the environment that serve as the prerequisite for exposure and the subsequent oral ingestion. In this study, we identified residential use as the primary source of the children’s pyrethroid exposure; however, this risk factor itself does not explain the age effect because age accounts for more variability in urinary metabolites than does residential use in the expanded mixed-effects model. One plausible explanation for this finding is that, as reported by their parents, many older children in this study were engaged in outside sports activities, such as swimming or playing tennis, in a neighborhood country club and parks during this sampling period. Pyrethroids used in those facilities may have led to increased pyrethroid exposure.

With the regulation change that led to the restricted use of OP pesticides in residential environments (U.S. EPA 1998), previously we were able to demonstrate that children are exposed to OP pesticides exclusively from their diets (Lu et al. 2006). Because fewer regulatory restrictions are imposed on the pyrethroid use, results from this study suggest that this same group of children are simultaneously exposed to pyrethroids via dietary intake and from their residential environments. Such diverse exposure patterns among children may pose a challenge for regulating pyrethroid insecticides as a group under the Food Quality Protection Act of 1996, which mandates the assessment of exposure in an aggregate manner. The implementation of the Food Quality Protection Act (1996) for pyrethroids could be even more problematic because of the difficulties of assessing cumulative risks resulting from pyrethroid exposures. Unlike OP or carbamate pesticides, pyrethroids do not appear to exhibit a single common toxicologic mechanism in humans.

Besides the limitations associated with this study that were discussed previously (Lu et al. 2006), the lack of environmental measures for pyrethroids makes it difficult to confirm the association between children’s exposure to pyrethroids and their residential use. Systematic quantification of pyrethroids and other nonpersistent pesticides, such as OP, in the environment, via soil, house dust, surface wipe, or personal breathing air collection, remains a daunting task. Substantial numbers of samples are needed to minimize the spatial and temporal variations, which is commonly associated with the measurements of nonpersistent pesticide exposures (Lu et al. 2004). The cost for analyzing such large numbers of environmental samples would compromise other aspects of a study with a limited research budget, such as reducing the number of participants or collecting less frequent biologic samples.

## Conclusion

We report the results from the first study of urban/suburban children’s longitudinal exposure to pyrethroid pesticides. In this study we found elevated urinary levels of pyrethroid metabolites associated with both residential pyrethroid use and diets. Pyrethroid use in the residential environment is a particular concern not only because exposures were routinely measured during the days when children consumed an organic diet but also because urine samples collected from this subgroup of children contained the highest levels of four pyrethroid metabolites. These findings provide an opportunity for intervention: The association between self-reported residential use of pyrethroids and the elevated pyrethroid metabolite levels found in the children can be broken by either minimizing the use of pyrethroids in the residential environment or eliminating children’s possible contact with treated areas or objects.

## Figures and Tables

**Table 1:** LODs and the number of urine samples [no. (%)] above or below the LODs for five common pyrethroid metabolites for 724 urine samples collected from 23 children for a 15-day study period.

	PBA	FPBA	*cis*-DCCA	*trans*-DCCA	DBCA
Detected	596 (82)	15 (2)	252 (35)	517 (71)	14 (2)
< LOD	23 (3)	41 (6)	22 (3)	50 (7)	15 (2)
Nondetected	105 (15)	668 (92)	450 (62)	157 (22)	695 (96)
LOD (μg/L)	0.1	0.2	0.2	0.4	0.1

**Table 2:** Descriptive statistics of DVWA concentrations (μg/L) of pyrethroid insecticide metabolites for 724 urine samples collected from 23 children for a 15-day study period.

	PBA	FPBA	*cis*-DCCA	*trans*-DCCA	DBCA
Mean ± SD	1.22 ± 2.4	0.02 ± 0.2	0.33 ± 1	1.24 ± 2.6	0.004 ± 0.02
Median	0.45	0	0	0.38	0
Geometric mean	0.58	0.08	0.4	0.54	0.05
Range	(0–25)	(0–3.5)	(0–15)	(0–25)	(0–0.1)
Percentiles					
10th	0.01	0	0	0	0
25th	0.22	0	0	0.14	0
75th	0.97	0	0.33	0.99	0
90th	2.85	0.05	0.90	3.37	0

**Table 3:** Self-reported use of pyrethroid pesticides in the households by the parents, and the number of days in which the metabolite concentrations of pyrethroid pesticides in their children’s urine samples exceeded the median DVWA levels for the respective pyrethroid metabolites.

			No. of days DVWA exceeded the median level^a^				
Child’s age(years)	Pyrethroids used	Location of use	PBA	FPBA	*cis*-DCCA	*trans*-DCCA	DBCA^b^
10	Ortho/carpenter ants (permethrin)	Home	3	8	1	2	0
8	Green light (permethrin/other pyrethroids)	Garden	15	4	7	15	11^c^
7	Terminix (permethrin)	Crawl space	10	1	3	9	3^c^
6	Pyrethroids E.C. (deltamethrin)	Deck	5	0	1	5	0
8	Hot Shot fogger (tetramethrin/permethrin)	Home	16	2	16	16	0
4	RID (furniture/bedding) (permethrin)	Beds	7^c^	1	6^c^	6^c^	0
11	Hartz (pyrethrin piperonyl butoxide)	Carpet	16	1	16	16	2^c^
	RID (pyrethrin)	Dog					
	Hartz control (allethrin)	Cat					

a Each child has a total of 16 days of DVWA concentration for each of the pyrethroid metabolites. Median levels for the DVWA concentration of five pyrethroid metabolites are given in Table 2.

b All urine samples with detectable levels were collected from children listed in this table.

c One urine sample has the highest level of the respective pyrethroid metabolite among the 724 urine samples collected.

**Table 4:** Selected SPSS results of a linear mixed-effects model for the DVWA of PBA and *trans*-DCCA concentrations (μg/L) in 23 children’s urine samples collected over a 15-day study period.

		PBA			*trans*-DCCA		
Source	df	Sum of squares	Mean square	*F*-value (Pr > *F*)^b^	Sum of squares	Mean square	*F*-value (Pr > *F*)
Residential pyrethroids use	1	9.6	9.6	19.6 (< 0.001)	5.4	5.4	15.4 (< 0.001)
Diets	1	2.0	2.0	4.0 (0.047)	1.9	1.9	5.4 (0.021)
Error	356	175.4	0.5		124.8	0.4	

Abbreviations: df, degrees of freedom; Pr, probability.

**Table 5:** DVWA concentrations of PBA and *trans*-DCCA in urine samples of 23 children over a 15-day study period, compared by diets, residential pyrethroids use, and age.

Source	PBA Mean (median)	*trans*-DCCA Mean (median)
Diet		
Conventional	1.24 (0.49)*	1.25 (0.42)**
Organic	1.16 (0.36)*	1.21 (0.28)**
Residential pyrethroids use		
No	0.94 (0.39)^#^	0.94 (0.35)^#^
Yes	1.84 (0.6)*^#^*	1.91 (0.57)*^#^*
Age (years)		
3–7	0.69 (0.37)^#^	0.66 (0.35)*^#^*
8–11	1.91 (0.86)*^#^*	1.99 (0.72)*^#^*

* Significantly different (one-way ANOVA, *p* = 0.023).

** Significantly different (one-way ANOVA, *p* = 0.008).

# Significantly different (one-way ANOVA, *p* < 0.001).

**Table 6:** Selected SPSS results of an expanded linear mixed-effects model for the DVWA of PBA and *trans*-DCCA concentrations (μg/L) in 23 children’s urine samples collected over a 15-day study period.

		PBA			*trans*-DCCA		
Source	df	Sum of squares	Mean square	*F*-value (Pr > *F*)	Sum of squares	Mean square	*F*-value (Pr > *F*)
Residential pyrethroids use	1	4.6	4.6	10.5 (0.001)	2.5	2.5	7.9 (0.005)
Diets	1	1.8	1.8	4.2 (0.04)	1.9	1.9	5.9 (0.016)
Age (years)	1	18	18	40.9 (< 0.001)	11.7	11.7	36.7 (< 0.001)
Error	352	155.1	0.4		112.7	0.3	

Abbreviations: df, degrees of freedom; Pr, probability.
